# Supplementary material for: Cepharanthine Enhances MHC-I Antigen Presentation and Anti-Tumor Immunity in Melanoma via Autophagy Inhibition
Source: Cells. 2025 Aug 9;14(16):1231. doi: 10.3390/cells14161231 (PMC12384187; doi:10.3390/cells14161231)
Supplement: Supplementary file 1 [file cells-14-01231-s001.zip › cells-3772200-supplementary.pdf]

Supplementary Materials for

Cepharanthine Enhances MHC-I Antigen Presentation and Anti-Tumor Immunity in Melanoma via Autophagy Inhibition

He Luo, Dan Chen, Jing Zhou, Dingye Wang, Qingsong Du, Qianwei Cai, Sixian lv, Xu Zhao, Guangxian Zhang, Yuhui Tan, He Jin, Xiaoyi Liu, Hua Yi and Jieying Guan

Corresponding author: 050156@gzucm.edu.cn (J.G.); 020693@gzucm.edu.cn (H.Y.); liuxiaoyi@gzucm.edu.cn (X.L.).

The PDF file includes:

Materials and Methods
Figure S1
Figure S2

Materials and Methods

CCK8 Assay for Cell Viability

To assess cell proliferation, B16 melanoma cells were seeded into 96-well plates at a density of 5×10^3 cells per well and incubated overnight to allow for cell attachment. Cells were then treated with increasing concentrations of CEP (0, 1.25, 2.5, and 5 μM) for 24 hours. Cell viability was measured using the Cell Counting Kit-8 (CCK-8; Dojindo Laboratories, Japan) following the manufacturer's instructions. Briefly, 10 μL of CCK-8 reagent was added to each well, and plates were incubated for 2 hours at 37 °C in a 5% CO_2 atmosphere. Absorbance at 450 nm was recorded using a multimode microplate reader. Relative cell viability was calculated as a percentage of the absorbance value relative to that of the untreated control group.

Supplementary Figures

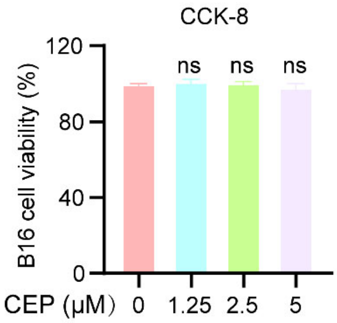

Figure S1. CEP does not induce cytotoxicity in B16 melanoma cells. B16 melanoma cells were treated with increasing concentrations of CEP (0, 1.25, 2.5, and 5 μM) for 24 hours. Cell viability was evaluated using the CCK-8 assay. Results showed no significant cytotoxic effects of CEP at the tested concentrations. ns, not significant.

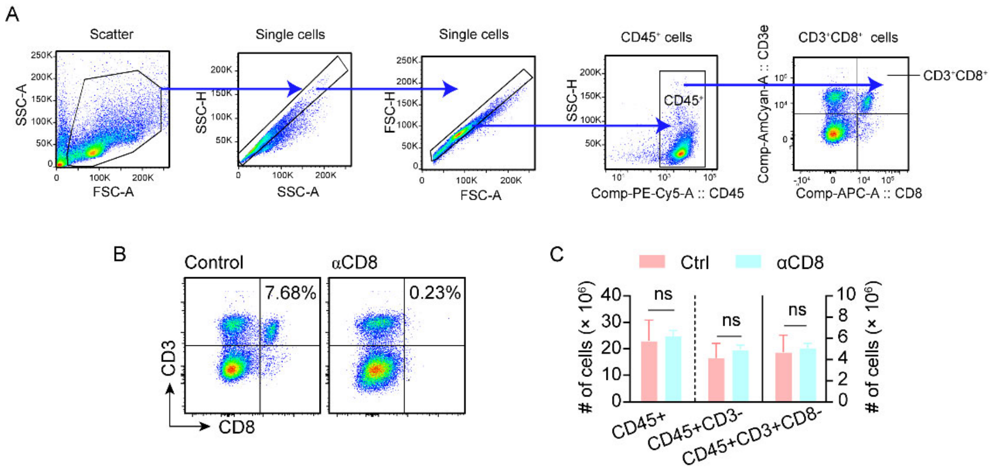

Figure S2. Evaluation of CD8⁺ T cell depletion efficiency following αCD8 antibody treatment. (**A**) Flow cytometry gating strategy for CD8⁺ T cell identification. Singlet events were gated from splenocytes, followed by sequential selection of CD45⁺ leukocytes, and then CD3⁺ CD8⁺ T cells. (**B**) Comparison of CD8⁺ T cell frequencies in control and αCD8-treated mice. Flow cytometric analysis demonstrated efficient depletion of CD3⁺ CD8⁺ T cells in αCD8-treated mice (control: 7.68% vs αCD8: 0.23%). (**C**) Quantitative analysis of major splenic immune cell subsets. Selective depletion of CD8⁺ T cells was confirmed, with no significant changes in total CD45⁺ cells, CD45⁺ CD3⁻ cells, or CD45⁺ CD3⁺ CD8⁻ populations. ns, not significant.
